# Ligation Tunes Protein Reactivity in an Ancient Haemoglobin: Kinetic Evidence for an Allosteric Mechanism in *Methanosarcina acetivorans* Protoglobin

**DOI:** 10.1371/journal.pone.0033614

**Published:** 2012-03-27

**Authors:** Stefania Abbruzzetti, Lesley Tilleman, Stefano Bruno, Cristiano Viappiani, Filip Desmet, Sabine Van Doorslaer, Massimo Coletta, Chiara Ciaccio, Paolo Ascenzi, Marco Nardini, Martino Bolognesi, Luc Moens, Sylvia Dewilde

**Affiliations:** 1 Dipartimento di Fisica, Università degli Studi di Parma, Parma, Italy; 2 NEST, Istituto Nanoscienze-CNR, Pisa, Italy; 3 Department of Biomedical Sciences, University of Antwerp, Antwerp, Belgium; 4 Dipartimento di Biochimica e Biologia Molecolare, Università degli Studi di Parma, Parma, Italy; 5 Department of Physics, University of Antwerp, Antwerp, Belgium; 6 Dipartimento di Scienze Cliniche e Medicina Traslazionale, Università di Roma Tor Vergata, Roma, Italy; 7 Consorzio Interuniversitario di Ricerca in Chimica dei Metalli nei Sistemi Biologici, Bari, Italy; 8 Dipartimento di Biologia, Università Roma Tre, Roma, Italy; 9 Dipartimento di Scienze Biomolecolari e Biotecnologie and CIMAINA, Università degli Studi di Milano, Italy; University of South Florida College of Medicine, United States of America

## Abstract

Protoglobin from *Methanosarcina acetivorans* (*Ma*Pgb) is a dimeric globin with peculiar structural properties such as a completely buried haem and two orthogonal tunnels connecting the distal cavity to the solvent. CO binding to and dissociation from *Ma*Pgb occur through a biphasic kinetics. We show that the heterogenous kinetics arises from binding to (and dissociation from) two tertiary conformations in ligation-dependent equilibrium. Ligation favours the species with high binding rate (and low dissociation rate). The equilibrium is shifted towards the species with low binding (and high dissociation) rates for the unliganded molecules. A quantitative model is proposed to describe the observed carbonylation kinetics.

## Introduction

An extensive search for putative globin genes in sequenced genomes within all kingdoms of Life revealed the presence of 3 globin lineages that probably evolved from a flavo-haemoglobin-like single-domain ancestral protein [1]. One lineage comprises the chimeric flavo-haemoglobins and related single domain globins. A second lineage includes the 2/2 globins. The third lineage consists of two-domain globin-coupled sensors, single-domain sensor globins, and a small number of related single-domain protoglobins (Pgbs) [2], [3], [4], [5]. Up to now, Pgbs have been identified in both *Archaea* and *Bacteria* [1], [5]. Their function is as yet unknown.

The crystal structure of a Pgb from *Methanosarcina acetivorans* (*Ma*Pgb), a strictly anaerobic methanogenic *Archaea*, was recently reported [6]. Phylogenetic analysis indicated that methanogenic *Archaea* are ancient organisms [7] that are located among the deepest branching chemoautotrophs on the tree of life, originating perhaps 3.8–4.1 billion years ago [8]. The *Methanosarcinae* are metabolically and physiologically the most versatile methanogens, being the only species endowed with all three known pathways for methanogenesis [9]. *Methanosarcina acetivorans* can metabolize carbon monoxide into methane and acetate using a phosphotransacetylase and an acetate kinase. This pathway is surprisingly simple and has been proposed to be the first metabolic pathway used by primordial microbes [10], [11]. One possibility is that Pgbs play a yet unclear role in the CO metabolism of these ancient organisms, thus making the study of CO binding to and dissociation from *Ma*Pgb potentially relevant from the physiological viewpoint. Additionally, the stability of CO complexes and the absence of oxidation reactions allow extensive kinetic investigations, as shown for many globins [12].

The three-dimensional structure of the *Ma*Pgb evidenced several peculiar topological features [6]. Unlike all other known globins, Pgb-specific loops and the amino-terminal extension completely bury the haem within the protein matrix. The haem moiety can reversibly bind diatomic ligands, such as O_2_, CO and NO, which are likely to access the haem through the Pgb-specific apolar tunnels connecting the solvent to the distal site from locations at the B/G and B/E helix interfaces. In addition, a core cavity of about 75 Å^3^ is located between the distal and proximal haem sides. The cavity hosts four mutually hydrogen-bonded water molecules. Residues lining the protein tunnels and the inner cavity are conserved in all known Pgbs. Finally, *Ma*Pgb assembles into a homodimer with a well-defined intermolecular contact surface.

Early rapid mixing experiments on the wild-type protein have shown that CO (and O_2_) binding to *Ma*Pgb is a biphasic process [6]. This feature raises the question whether the heterogeneity arises from the existence of two molecular populations in equilibrium or if it is due to the two different entry pathways through the hydrophobic tunnels. In addition, it is critical to assess to what extent the protein dynamics and ligation introduces a regulation of the protein function, possibly associated with intermolecular interactions. Recent results from Molecular Dynamics suggest that ligation influences the opening of tunnel 1, thus potentially regulating the exchange rates of reactants between the reaction site and the solvent [13].

Using vibrational and time-resolved spectroscopy, we show herein that *Ma*Pgb exists in two distinct conformations with different affinity for CO, in an equilibrium influenced by the ligation state of the protein. This mechanism is mediated by protein dynamics and suggests that in this ancient protein an allosteric regulation of the affinity towards ligands has been developed. The homotropic allosteric control of affinity may be relevant for a bimolecular reaction towards the yet unknown physiological substrates of the protein.

## Methods

### Recombinant expression and purification of MaPgb

All experiments reported herein were performed on the *Ma*Pgb mutant bearing the C(E20)101S mutation (hereafter termed simply *Ma*Pgb*) produced for crystallization purposes. The mutation was introduced in *Ma*Pgb using the QuickChange™ site-directed mutagenesis method (Stratagene), as described earlier [14]. *Ma*Pgb* was expressed in *Escherichia coli* BL21(DE_3_)pLysS cells (Invitrogen) and collected as described previously [14]. Alternatively, cells were resuspended in 50 mM Tris–HCl pH 8.0, 5 mM EDTA, 0.5 mM DTT and 1 mM phenylmethylsulphonyl fluoride.

For the extraction and purification of recombinant proteins, the cells were exposed to three freeze-thaw steps and sonicated until completely lysed. Inclusion bodies were washed twice with 50 mM Tris–HCl (pH 8.0), 5 mM EDTA and 2% sodium deoxycholate, washed once with pure water and solubilized by incubation in 100 mM Tris-NaOH (pH 12.0) and 2 M urea. After an incubation period of 30 min at room temperature and centrifugation at 10,700 *g* for 20 min at 4°C, *Ma*Pgb* was refolded by adding a 1.5 molar excess of haemin (7.6 mM in 10 mM NaOH). After an incubation period of another 10 min at room temperature, the pH was adjusted to 8.5 with HCl. The solution was then diluted into 5 volumes of distilled water and finally dialysed at 4°C against gel filtration buffer (50 mM Tris-HCl pH 8.5, 150 mM NaCl and 0.5 mM EDTA). Final purification was performed by gel filtration using a Sephacryl S200 column equilibrated in this buffer.

### Analytical gel filtration experiments

Analytical gel filtration experiments were performed to determine the quaternary structure of *Ma*Pgb* in solution. Therefore, 5 globin standards (human haemoglobin (HbA) - purified according to Rivetti et al. [15], human cytoglobin (Cygb) - recombinantly expressed and purified according to Dewilde et al. [16], bovine serum albumin (BSA), horse heart myoglobin (Mb) and cytochrome *c* (cyt *c*) – purchased from Sigma) and *Ma*Pgb* were passed through a Superdex G75 column (2 cm×30 cm) equilibrated at room temperature with 50 mM Tris-HCl pH 8.5, 150 mM NaCl and 0.5 mM EDTA. The flow rate was 0.2 ml/min, and protein elution was monitored at 280 nm and 412 nm. To determine whether protein concentration influences the degree of quaternary assembly of *Ma*Pgb*, different concentrations of *Ma*Pgb* were analyzed (3 µM, 12.5 µM, 50 µM and 100 µM).

### Dynamic light scattering

Experiments were conducted using a Protein Solutions DynaPro 99 instrument with a DynaProMSTC200 microsampler (Protein Solutions, Charlotville, VA). Protein concentration was 22 µM (10 mM Tris-HCl, pH 7.0, 150 mM NaCl). Acquisition was performed at 10°C with Dynamics 5, 30 scan, 30 sec/each scan. Data analysis was performed with the Dynamics 6 software.

### Resonance Raman measurements

Resonance Raman spectra of *Ma*Pgb* were recorded with a Dilor XY-800 Raman scattering spectrometer consisting of a triple 800 mm spectrograph operating in low-dispersion mode and using a liquid nitrogen-cooled CCD detector. The excitation source for all spectra is a Kr^+^ laser (Spectra-Physics Beamlok 2060) operating at 413.1 nm. The spectra were recorded at room temperature. The *Ma*Pgb* solution was stirred at 500 rpm to avoid local heating and photochemical decomposition in the laser beam. Twelve spectra (10–240 s recording time each) were acquired to allow for the removal of cosmic ray spikes. This was done by eliminating the lowest and highest data points for each frequency value and averaging the remaining values. Laser power was in the 0.5 to 150 mW range.

### Stopped flow experiments

Kinetics of CO binding to and dissociation from *Ma*Pgb* were carried out in 100 mM potassium phosphate buffer pH 7.0 at 20°C employing a thermostatted stopped-flow apparatus (Applied Photophysics, Salisbury, UK) with a dead time of 1 ms. For the CO association kinetics, a 10 µM deoxy Fe^2+^ *Ma*Pgb* solution was mixed with a solution equilibrated with nitrogen/CO mixtures at different CO partial pressures. Sodium dithionite was added anaerobically to both the protein solution and the CO solutions to a final concentration of 2 mM. *Ma*Pgb* carbonylation was monitored at 418 nm.

The CO dissociation was induced by mixing a CO*Ma*Pgb* solution (10 µM) with a 180 µM NO solution. The protein solution was degassed, reduced with sodium dithionite (2 mM), exposed to CO and then extensively treated in nitrogen flux to remove excess CO. The NO solution was prepared by anaerobically dissolving the NO donor MAHMA NONOate (Sigma Aldrich) in a previously degassed buffer solution. The NO donor solution was left equilibrating for about 30 minutes, a time much longer than that needed for the NO release, a process occurring with a time constant of about 3 min. The exact concentration of NO was determined by titration with human deoxy-HbA under anaerobic conditions. The displacement of CO by NO was monitored through the absorbance changes at 421 nm.

O_2_ dissociation was induced by mixing a O_2_ *Ma*Pgb* solution with a degassed sodium dithionite solution (10 mM final concentration) at pH 7.0 and 20°C. A second mixing was then performed with CO containing solutions, at several CO concentrations, and different delays along the O_2_ dissociation progress curve. This allowed to induced CO binding to *Ma*Pgb* with various fractional ligand saturation values.

### Nanosecond laser flash photolysis

For nanosecond laser flash photolysis experiments, the Fe^3+^ *Ma*Pgb* stock solution was diluted with a 100 mM phosphate buffer at pH 7.0 to a final concentration of 50 µM, equilibrated in either 1 atm or 0.1 atm CO and anaerobically reduced with sodium dithionite (2 mM). The experimental setup was described in detail elsewhere [17]. Photolysis of CO *Ma*Pgb* was obtained using the second harmonic (532 nm) of a Q-switched Nd:YAG laser (HYL-101, Quanta System). The cw output of a 75 W Xe arc lamp was used as probe beam, a 5 stages photomultiplier (Applied Photophysics) for detection and a digital oscilloscope (LeCroy LT374, 500 MHz, 4 GS s^−1^) for digitizing the voltage signal. A spectrograph (MS257 Lot-Oriel) was used to select the monitoring wavelength (436 nm) and to remove the residual stray light from the pump laser. The sample holder is accurately temperature controlled with a Peltier element, allowing a temperature stability of better than 0.1°C.

Time-resolved difference absorbance spectra were measured using a gated intensified CCD (Andor Technology, iStar, 1024×1024 pixels used in full vertical binning mode), coupled to the spectrograph. Time-resolved spectra consist of photoproduct minus CO*Ma*Pgb* difference spectra at 70 logarithmically spaced time delays following photodissociation, from 10 ns after the laser flash to 10 ms. Spectra are obtained by averaging 100 single shot signals at each delay.

### Encapsulation *in silica* gel

Encapsulation of *Ma*Pgb* in silica gel was carried out in both the deoxy state (*Ma*Pgb* gels) and in the carboxy state (CO*Ma*Pgb* gels) using a previously described protocol [18]. For *Ma*Pgb* gels, the equilibration with CO was carried out immediately before the flash photolysis experiments.

### Data analysis of the ligand rebinding kinetics

In order to highlight the relative roles of the kinetic phases, lifetime distributions associated with the observed CO rebinding kinetics were evaluated using the program MemExp [19], [20]. The program uses a maximum entropy method (MEM) and either nonlinear least-squares (NLS) or maximum likelihood (ML) fitting to analyze a general time-dependent signal in terms of distributed and discrete lifetimes. A distribution of effective log-lifetimes, g(log(τ)), was extracted from the data without restriction to any functional form and its effectiveness has already been tested on several other globins [21], [22], [23].

Time-resolved difference spectra were analyzed by singular value decomposition (SVD) [17], [24], [25] using MATLAB (The Mathworks, Inc., Natick, MA).

We have followed a minimal model to describe the rebinding kinetics (Scheme 1 within Discussion). The differential equations corresponding to Scheme 1 were solved numerically within Matlab using the ODE15s function, and the rate constants optimized with the package Minuit (CERN) to obtain a least-squares best fit to the experimental data [17], [21], [22]. In order to improve retrieval of microscopic rate constants, data at the same temperature but different CO concentrations (1 and 0.1 atm) were simultaneously analyzed. The global analysis was repeated at different temperatures between 10 and 40°C. As a further check, the global analysis procedure was applied also to fit simultaneously CO binding kinetics from stopped flow and flash photolysis at 20°C. Activation parameters for the microscopic rate constants were determined from the resulting linear Eyring plots (Table 1).

**Table 1:** Microscopic rate constants for *Ma*Pgb* from the fit of the flash photolysis data, at 20°C.

		solution		CO*Ma*Pgb* gel		(CO+*Ma*Pgb*) gel
	*k*	*ΔG* ^‡^(kcal/mol)	*k*	*ΔG* ^‡^(kcal/mol)	*k*	*ΔG* ^‡^(kcal/mol)
*k*_1_ (10^5^ s^−1^)	1.35	10.51±0.1	1.35	10.5±0.02	1.35	10.52±0.02
*k*_−1_(10^5^ s^−1^)	4.55	9.8±0.1	4.55	9.8±0.1	4.5	9.8±0.02
*k*_2_(10^5^ s^−1^)	0.6	10.9±0.2	0.4	11.1±0.4	1	10.5±0.6
*k*_−2_ (10^4^ s^−1^)	2.5	11±1	1.4	11.7±0.1	2.5	11.49±0.03
*k*_3_ (10^5^ s^−1^)	0.6	10.9±0.2	0.4	11.0±0.7	1.1	10±2
*k_−_*_3_ (10^4^ s^−1^)	2.0	11.5±1.1	1.4	11.7±0.4	2.5	11.48±0.03
*k_in,r_* (10^7^ M^−1^ s^−1^)	7.8	6±2	7.2	6±3	6.9	6±2
*k_in,t_* (10^7^ M^−1^ s^−1^)	3	7±3	1	6.8±1	1	7±2
*k_out_* ** (10^8^ s^−1^)	1.5	6±1	1.4	6±4	1	6±3
*k_g,r_* (10^7^ s^−1^)	5.5	6.9±0.1	5.3	7.1±0.1	3.8	7.16±0.02
*k_g,t_* (10^6^ s^−1^)	6	8.2±0.1	3.8	8.4±0.1	3.8	8.53±0.02
*k_d,r_* (10^−2^ s^−1^)	4.4	19.6±0.1	4.4	19.6±0.1	4.4	19.65±0.02
*k_d,t_* (10^−2^ s^−1^)	8.4	19.1±0.1	8.3	19.0±0.1	8.4	19.06±0.02
*k*_c_ (10^7^ s^−1^)*	1	8.0±0.1	1	8.7±0.7	1	7.95±0.02
*k_−_*_c_ (10^7^ s^−1^)*	1	8.0±0.1	0.7	8.0±0.1	0.9	7.95±0.02
*k*_d_ (10^7^ s^−1^)*	1	8.0±0.1	1	8.8±0.9	0.6	9.6±0.02
*k*_−d_ (10^7^ s^−1^)*	1	8.0±0.1	0.7	7.9±0.1	1.1	7.89±0.02

Activation free energies were estimated from the linear Eyring plots for each rate constant *k*_i_ in the temperature range 10–40°C.

* The value of these rates and their associated temperature dependence are difficult to estimate given the small amplitude of the processes.

** the present analysis did not allow to appreciate a difference in *k_out_* for the two conformations.

The numerical reliability of each parameter was defined by evaluating the dependence of the fitness function on changes of the parameter, assuming the others as constant. *k*_−1_≈; *k*_2_ , *k*_−2_≈10%; *k*_3_ , *k*_−3_≈10%.

The rate equations for CO binding are very poorly sentitive to *k_d,r_* and *k_d,t_*, which are held constant to the values determined by the following equation: *k_OFF,1/2_* = *k_d,r/t_*×*k_out_*/(*k_out_*+*k_g,r/t_*).

## Results

### Electronic absorption spectroscopy

**Figure 1:**
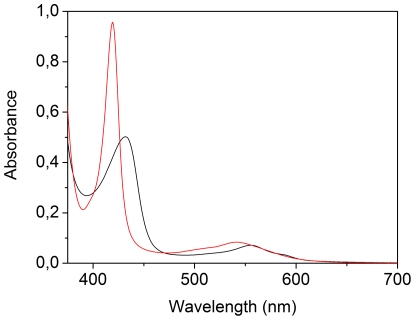
Electronic absorption spectra of *Ma*Pgb*. Dithionite-reduced Fe^2+^ deoxy form (black line) and the CO-bound form (red line). The protein concentration was 20 µM. Spectra were measured with an optical pathlength of 0.2 cm.

Figure 1 shows the UV-VIS absorption spectrum of *Ma*Pgb*, in the dithionite-reduced Fe^2+^ deoxy form (black line) and the CO-bound Fe^2+^ form (red line).

The Soret-band of deoxy Fe^2+^ *Ma*Pgb* exibits a band at 432 nm, with a Q-band at 556 nm and a shoulder at 588 nm, typical of a ferrous pentacoordinated high-spin form. The CO-form has the Soret-band at 419 nm, and a Q-band at 542 nm with a shoulder at ∼510 nm. This spectrum is comparable to that of the CO-form of wild type *Synechocystis* Hb [26].

### Resonance Raman spectra of Fe^2+^ *Ma*Pgb* and CO*Ma*Pgb*

**Figure 2:**
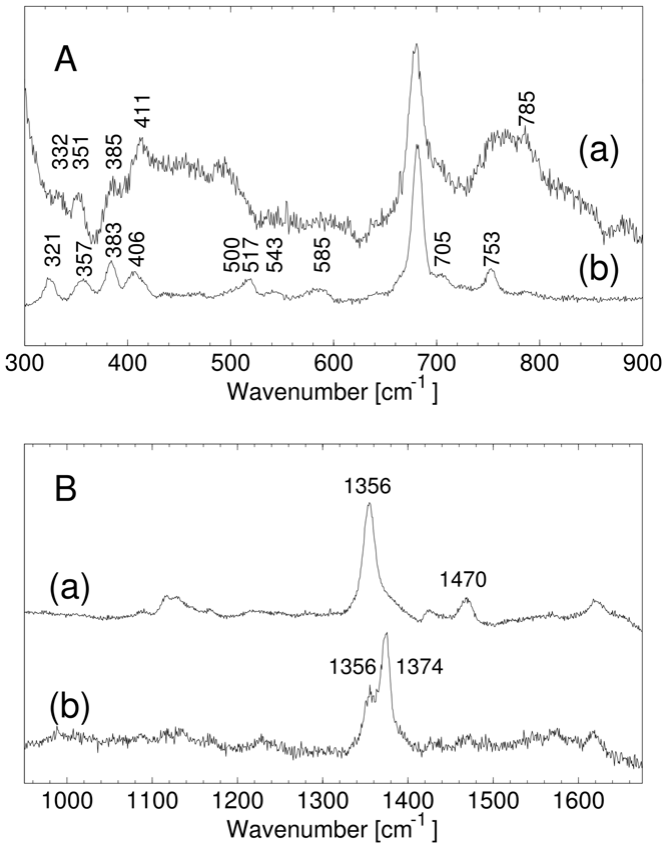
Resonance Raman spectra of *Ma*Pgb*. Low-frequency (**A**) and high-frequency (**B**) region of the RR spectrum of Fe^2+^ *Ma*Pgb* (a) CO*Ma*Pgb*. The excitation wavelength was 413.1 nm, laser powers employed were (a) 100 mW, and (b) 1 mW.

Figure 2 shows the resonance Raman (RR) spectra of *Ma*Pgb*, in the deoxy Fe^2+^ (Fe^2+^ *Ma*Pgb*) and the CO-bound (CO*Ma*Pgb*) forms.

In the high-frequency region of the RR spectra of Fe^2+^ *Ma*Pgb* (Figure 2B), the marker bands are located at *ν_4_*  = 1356 cm^−1^, and *ν_3_* = 1470 cm^−1^. This is typical of a pentacoordinated high-spin ferrous haem [27]. No clear maximum can be observed in the *ν_2_*-region of Fe^2+^ *Ma*Pgb*.

The RR spectrum of CO*Ma*Pgb* recorded at 1 mW laser power shows two bands in the *ν_4_* region of the spectrum, one band at 1374 cm^−1^, corresponding to the CO-bound Pgb*, and another band at 1356 cm^−1^, indicating the presence of deoxy Fe^2+^ haem. The presence of ferrous haem in the RR spectrum of CO*Ma*Pgb* indicates that, even at 1 mW laser power, part of the haem-bound CO is photolyzed. This is confirmed by the fact that at higher laser powers the fraction of deoxy Fe^2+^ haem increases (Figure S1).

In the low-frequency part of the spectrum of partially photolyzed CO*Ma*Pgb*, a band can be observed in the ν_Fe-His_ region at 219 cm^−1^ (Figure S1). This band depends on the laser power and can thus be assigned to the ν_Fe-His_ mode. The frequency is comparable to that of sperm whale myoglobin (Mb) (220 cm^−1^) and barley Hb (219 cm^−1^) [28]. The value of this mode indicates that the proximal histidine in penta-coordinated deoxy ferrous *Ma*Pgb* has an uncharged imidazole character [28] and the haem is tightly bound to the proximal histidine [29]. The frequency is quite close to that of the R-state in human Hb (222 cm^−1^), indicating a relatively unstrained proximal histidine.

In the low-frequency part (Figure 2A) of the RR spectra, the propionate bending mode of Fe^2+^ *Ma*Pgb* is found at 385 cm^−1^, indicating a very strong H-bonding interaction. Similarly, this mode lies at 383 cm^−1^ in CO*Ma*Pgb*. The vinyl bending-mode lies at 411 cm^−1^ in the deoxy ferrous *Ma*Pgb*, and is comparable to that of deoxy *Lucina pectinata* HbI [30]. It shifts to a lower wavenumber (406 cm^−1^) upon CO ligation.

For CO-bound ferrous haem proteins, the low-frequency region of the RR spectrum contains a number of bands that belong to the bound CO ligand. The frequency of these modes reflects whether the ligand experiences an open haem pocket, with little interaction with amino-acid residues, or a closed haem pocket in which it interacts more strongly with the residues in the haem-pocket. The RR spectrum of CO*Ma*Pgb* (Figure 2A), reflects a large degree of heterogeneity in the haem-pocket. Several ν_Fe-CO_ modes can be found starting at ∼500 cm^−1^, with peaks at 517 cm^−1^ and 543 cm^−1^ (identification of CO-related bands was done via photolysis experiments). 543 cm^−1^ is a very high frequency for a Fe-CO stretching mode. A similar frequency was observed in *Ascaris* Hb [31] and horseradish peroxidase (in peroxidases it is attributed to the imidazolate character of the proximal histidine, which is not a possible explanation for *Ma*Pgb* considering the ν_Fe-His_ frequency). The lowest frequency at approximately 500 cm^−1^ corresponds to a fairly open haem pocket with little interaction between the CO and nearby amino-acid residues. The higher frequency of 517 cm^−1^ indicates strong electrostatic interactions between the haem bound CO and the distal side of the haem pocket [30]. Note the similarity with human neuroglobin, where also three CO-binding conformations were found to coexist, with frequencies for ν_Fe-CO_ of 494 cm^−1^ (open conformation), 505 cm^−1^ (intermediate) and 521 cm^−1^ (closed) [32]. At present, the distal side residue(s), which is (are) responsible for the specific interactions with the bound CO, have not yet been identified. However recent crystallographic analyses on cyanide-bound ferric *Ma*Pgb* derivative, as the wild type form and specific mutants, showed that residue Trp(60)B9 and Tyr(61)B10 indeed change their side chain orientations and stabilize the haem-bound cyanide (Nardini et al., unpublished results).

The Fe-CO bending mode, δ(Fe-CO), can be observed at 585 cm^−1^. A similar bending mode was also observed in the RR spectrum of CO-bound *Ascaris* Hb [31]. In *Ascaris* Hb, a Fe-CO bending mode at 588 cm^−1^ was assigned to a conformer with a Fe-CO stretching mode at 543 cm^−1^. It should be noted that the δ(Fe-CO) band in *Ma*Pgb* is very broad and contains one or more different frequencies of the bending mode that correspond to the lower-frequency stretching modes.

### Oligomerization state analysis by gel filtration and Dynamic Light Scattering

Analytical gel filtration experiments were performed to assess the quaternary structure of *Ma*Pgb* in solution (expected molecular mass = 23 kD, as estimated from sequence analysis). Elution time for *Ma*Pgb* (Figure S2) corresponds to a molecular mass of 51 kDa, a value perfectly consistent with a dimeric assembly. *Ma*Pgb* elution time was the same for all tested concentrations (between 3 and 100 µM), indicating the presence of dimeric *Ma*Pgb* in solution at all protein concentrations in the range employed in this study. To check if the oxidation state of the haem iron atom influences the assembly of subunits, the experiment was repeated in the presence of 20 mM sodium dithionite. Results were identical, suggesting that protein assembly is independent of the oxidation state (data not shown).

Dynamic Light Scattering (DLS) analysis indicates that the *Ma*Pgb* sample is mostly monodisperse with 94.4% of the scattering mass having a hydrodynamic radius of 3.2 nm, corresponding to an estimated molecular mass of ∼50 kD.

### CO binding to and dissociation from MaPgb* by rapid mixing

**Figure 3:**
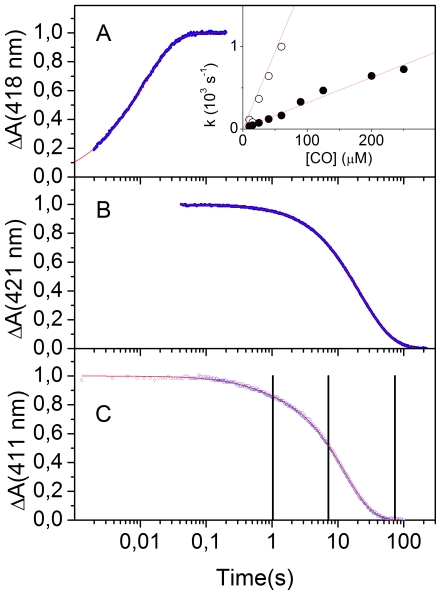
CO binding to and dissociation from *Ma*Pgb* from stopped flow. (**A**) Representative CO binding kinetics as measured through the normalized absorbance changes at 418 nm for *Ma*Pgb* in 0.1 M phosphate pH 7.0 at 20°C (blue dots). CO concentration was 25 µM. Solid red line is the non-linear least-squares fitting to a double exponential relaxation. Inset. CO concentration dependence of the apparent rate constants obtained from the fitting with a double exponential relaxation to the progress curves at 418 nm. (**B**) CO dissociation kinetics induced by mixing a CO*Ma*Pgb* solution with a NO-equilibrated solution as measured through the normalized absorbance changes at 421 nm for *Ma*Pgb* in 0.1 M phosphate pH 7.0 at 20°C (blue dots). Solid red line is the non-linear least-squares fitting to a double exponential relaxation. (**C**) O_2_ dissociation kinetics (blue circles, monitored at 411 nm) from Fe^2+^ O_2_ *Ma*Pgb* after addition of sodium dithionite (10 mM final concentration) at 20°C. The red solid line is the fit to a double exponential relaxation. Black vertical lines indicate the time at which mixing with CO was performed.

CO binding to deoxy Fe^2+^ *Ma*Pgb* (Figure 3A) occurs with a biphasic kinetics, which can be fit by a double exponential relaxation. From the slope of the CO concentration dependence of the apparent rate constants, we can estimate *k_ON,1_* = (1.87±0.08)×10^7^ M^−1^ s^−1^ (20%) and *k_ON,2_* = (3.1±0.2)×10^6^ M^−1^ s^−1^ (80%).

CO dissociation induced by NO displacement also shows a heterogenous kinetic pattern, suggesting multiple CO-bound forms, as also implicated by RR spectra (Figure 2). The experimental trace (blue dots, Figure 3B) can be best described by a bi-exponential relaxation, with observed off rates (amplitudes) *k*_OFF,1_ = (0.081±0.001) s^−1^ (33%) and *k*_OFF,2_ = (0.032±0.001) s^−1^ (67%). While showing a similar heterogeneity, the values reported here for *Ma*Pgb* differ from those reported previously, which were obtained for the wild type protein [6]. Recent computational analysis on the oxygenated form, suggest that the low value of the dissociation rates may be ascribed mostly to the strong distortion of the *Ma*Pgb* haem [33].

We then measured CO binding kinetics to partially oxygenated *Ma*Pgb*, prepared by mixing O_2_ *Ma*Pgb* and sodium dithionite. Figure 3C shows O_2_ dissociation kinetics from Fe^2+^ O_2_ *Ma*Pgb* after addition of sodium dithionite (10 mM final concentration). Vertical lines indicate time aging after sodium dithionite addition, before second mixing with buffer equilibrated at different CO concentrations. Second order rate constants for CO binding were found independent of the aging interval, *i.e.*, of the fractional saturation of the dimer. This rules out the possibility of a significant cooperativity for ligand binding to *Ma*Pgb*.

### CO rebinding kinetics after laser photolysis of *Ma*Pgb* in solution and in silica gels

**Figure 4:**
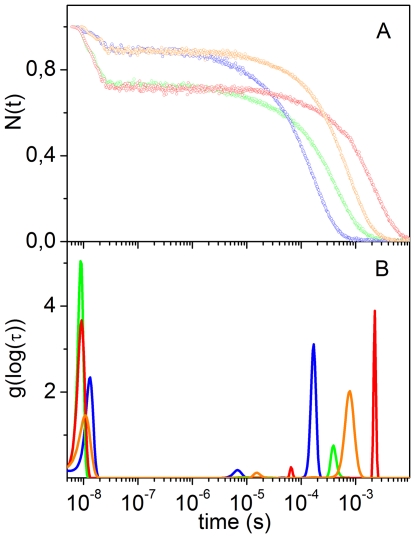
CO rebinding to *Ma*Pgb* after nanosecond laser photolysis. (A) CO rebinding kinetics to CO*Ma*Pgb* solutions at T = 10°C (green, 1 atm CO; red, 0.1 atm CO) and T = 30°C (blue, 1 atm CO; orange, 0.1 atm CO). (B) Lifetime distributions obtained from the MEM analysis of the CO rebinding curves shown in (A).

The CO rebinding kinetics following nanosecond laser photolysis of CO*Ma*Pgb* solutions were recorded as a function of CO concentration and temperature. Figure 4A reports the fraction of deoxy haems as a function of time after photolysis for selected conditions. The associated lifetime distributions retrieved from the MEM analysis are shown in Figure 4B.

The CO concentration dependence demonstrates the presence of two distinct processes in the CO rebinding curves. A faster rebinding phase, which is complete in less than 100 ns, shows no CO concentration dependence, and is ascribed to rebinding of photodissociated ligands from within the protein matrix (geminate rebinding). A slower phase, whose apparent rate constant is dependent on CO concentration, is associated with second order binding of ligands from the solvent phase.

The temperature dependence of the rebinding kinetics (Figure 4A) reveals that exit to the solvent is a thermally activated process, which leads to an enhanced probability of escaping from the protein matrix as the temperature is increased, resulting in a lower geminate recombination.

Analysis of the rebinding kinetics with the MEM (Figure 4B) affords lifetime distributions with several bands. The area and the position of the band with peak at ∼10 ns at 10°C are scarcely sensitive to CO concentration, suggesting an unimolecular nature for this process, associated with geminate rebinding. The area becomes smaller when temperature is increased, showing that the ligand escape from the distal pocket is thermally activated.

The lifetime distributions associated with the kinetics occurring on longer time scales evidence the presence of several concentration dependent bands, suggesting this phase comprises more than one step and/or reaction intermediate. A small amplitude band, with a remarkable temperature and ligand concentration sensitivity, is present under all conditions. It is centered at 7 µs at 30°C in the 1 CO atm distribution, and shifts to 15 µs in the 0.1 CO atm distribution. At the same temperature, a peak of larger amplitude is observed at about 170 µs at 1 atm CO, and shifts to ≈780 µs at 0.1 atm CO. Remarkably, the area of this second peak increases upon lowering the ligand concentration, suggesting that this slower step is favoured when the CO concentration is decreased.

To make this observation more quantitative, we have fitted the portion of the kinetic traces corresponding to bimolecular rebinding, with a sum of two exponential decay functions. Detailed results are reported as Figure S3. The analysis evidences the presence of two processes with lifetimes around 10^−6^ s and 10^−4^ s at 1 atm, and 10^−5^ s and 10^−3^ s at 0.1 atm, in agreement with the MEM results. Lifetimes and amplitudes are temperature dependent, with the faster phase decreasing, and the slower phase increasing, as the temperature is increased. The relative weight of the slower phase increases when lowering the ligand concentration (Figure S4).

The ligand concentration dependence of their amplitudes suggests that the two bimolecular rebinding phases observed in laser flash photolysis are associated with the presence of fast and slow rebinding conformations in equilibrium, whose relative concentrations are regulated by binding. The fast rebinding conformation is favoured by ligation, whereas unliganded proteins favour the slow rebinding conformation. In this view, when CO*Ma*Pgb* is photodissociated, the protein relaxes towards the slow rebinding state. As CO rebinding is made slower by reducing its concentration, the protein is allowed more time to relax towards the slow rebinding state, and the amplitude of the associated kinetic phase is accordingly increased. The shape of the rebinding kinetics is independent of the laser pulse energy, *i.e*. of the level of photolysis, and this strongly argues against the involvement of quaternary structural changes.

**Figure 5:**
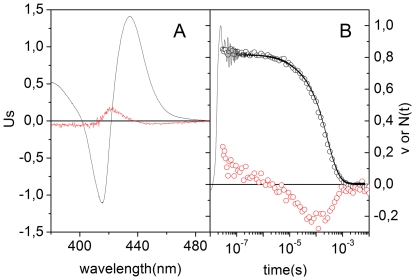
SVD analysis of time resolved spectra after nanosecond laser photolysis of CO*Ma*Pgb*. (A) Comparison of the first (U_1_, black line) and the second (U_2_, red line) components obtained from the SVD analysis on the time-resolved spectra measured for *Ma*Pgb* in solution. Spectra *U*_i_ are multiplied by the corresponding singular value *S*_i_. (B) Time courses of the amplitudes *V*_1_ (open black circles) and *V*_2_ (open red circles). The pressure of CO was 1 atm, and the spectra were measured at 20°C. The black curve in panel B is the absorbance change measured at 436 nm (in a single wavelength experiment) at the same CO pressure and temperature.

Time-resolved absorbance difference spectra following laser flash photolysis give further support to the occurrence of a conformational change upon photo-dissociation of the CO adduct. Figure 5 shows the first two meaningful spectral components and the time evolution of the amplitudes of each component, obtained from the SVD analysis of the time-resolved absorbance difference spectra (not shown), measured after nanosecond photolysis of CO*Ma*Pgb* solutions at room temperature. The spectral shape of the first SVD component *U*_1_ (autocorrelation = 0.99, *S*_1_ = 16.4) [24] corresponds to the carboxy-deoxy absorbance spectrum and is hence associated with rebinding. The second component *U*_2_ (autocorrelation = 0.91, *S*_2_ = 0.47) represents a minor contribution describing the changes in the shape of the absorption spectrum induced by sources other than ligand rebinding.

The time course of the amplitude *V*_1_ of the first component tracks the ligand rebinding kinetics and perfectly matches the kinetics measured at 436 nm, as shown in the right panel. At this wavelength the contribution of the second component (red spectrum in Figure 5) is almost negligible. The time course of the amplitude *V*_2_ of the second component suggests formation of a transient species on the microsecond time scale, which then decays at longer times, upon CO rebinding. The intermediate species is formed with a time extended kinetic process, not described by a single exponential relaxation (Figure S5).

Following the approach we successfully used to stabilize T and R states of human HbA [34], [35], [36], we have encapsulated *Ma*Pgb* in silica gels to trap fast and slow rebinding states. The fast rebinding conformation was trapped by encapsulating the CO complex of the protein (CO*Ma*Pgb* gels), whereas the slow rebinding species was stabilized by encapsulating deoxy *Ma*Pgb* (*Ma*Pgb* gels). CO was added to *Ma*Pgb* gels immediately before laser flash photolysis experiments (*Ma*Pgb* + CO gels). If the gel were capable of stabilizing the two extreme conformations, CO rebinding kinetics to CO*Ma*Pgb* gels and to *Ma*Pgb* + CO gels would (mostly) reflect affinity for CO of the two species.

**Figure 6:**
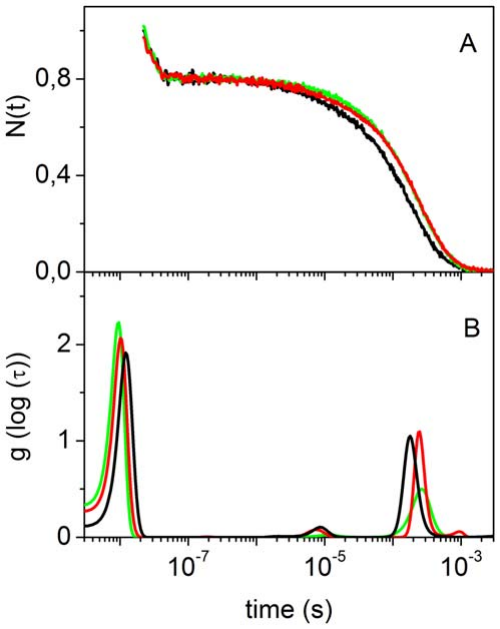
CO rebinding to *Ma*Pgb* gels after nanosecond laser photolysis. (A) Comparison between the CO rebinding kinetics after photolysis of CO*Ma*Pgb* solutions (red curve), CO*Ma*Pgb* gels (black curve) and *Ma*Pgb* + CO gels (green curve). All displayed curves were measured at *T* = 20°C and 1 atm CO. (B) Lifetime distributions retrieved from fitting with the MEM in panel A (color code as in panel A).

Figure 6 compares the CO rebinding kinetics for *Ma*Pgb* in solution and gels, and demonstrates that the gel structure has indeed appreciable, although small, effects on the ligand rebinding kinetics. In the selected conditions, the geminate phase is essentially identical both in amplitude and rate, while the most significant differences appear in the bimolecular phase. For CO*Ma*Pgb* gels, second order rebinding kinetics is a faster process, while for *Ma*Pgb* + CO gels rebinding is slower. Rebinding to *Ma*Pgb* solutions occurs with an intermediate progress curve, in which a clearly biphasic trend can be recognized.

The MEM analysis for CO rebinding to *Ma*Pgb* + CO and to CO*Ma*Pgb* gels yields lifetime distributions with bands at ∼10 ns, associated with geminate recombination, and two bands centered at ∼10 µs and ∼200 µs, whose position is dependent on CO concentration (Figure S6), suggesting they are associated with distinct bimolecular processes.

The area of the fast bimolecular rebinding peak for CO*Ma*Pgb* gels is around 5 times the area under the corresponding band for *Ma*Pgb* + CO gels, whereas the area of the slow bimolecular rebinding peak is almost independent on protocol of encapsulation. The lifetime distribution for CO rebinding to *Ma*Pgb* in solution shows features of both CO*Ma*Pgb* and *Ma*Pgb* + CO gels.

The gel results support the hypothesis outlined above that the biphasic second order rebinding kinetics derives from the existence of two (fast and a slow rebinding) conformations in equilibrium in solution. The ligand biases the equilibrium towards the fast binding species; its absence pulls the equilibrium to the slow binding species side.

## Discussion

Gel filtration experiments suggest that *Ma*Pgb* exists as a dimer in solution at the concentrations employed in all of the experiments reported herein (Figure S2). The dimerization state of the protein in solution is further indicated by Dynamic Light Scattering analysis. Stability of the quaternary assembly is suggested by the strong interactions and the large contact surface between the two molecules in the crystallographic unit cell, which comprises mostly residues belonging to the G- and H-helices, to the H_0_-helix, partly to the Z-helix and to the BC and FG hinges [6]. The buried interface calculated on the dimer derived from the *Ma*Pgb*crystal structure (pdb-code 2VEB) is of 1847 Å^2^, with a free energy of dissociation ΔG^diss^ = 24.8 kcal/mol (with 24 potential hydrogen bonds and 10 salt bridges across the interface) as calculated by the program PISA (http://www.ebi.ac.uk/msd-srv/prot_int/pistart.html) [37]. ΔG^diss^ corresponds to the free energy difference between dissociated and associated states and, therefore, assemblies with ΔG^diss^>0 are thermodynamically stable. The dimeric structure of *Ma*Pgb* is also suggested by the presence of an identical quaternary assembly in a different crystal form (pdb-code 2VEE, four dimers in the asymmetric unit) and a similar dimeric structure in the closely related globin coupled sensors [4]. In particular, analysis of the dimeric interface of the globin domain of the GCS from *B. Subtilis* (pdb-code 1OR4 [38]), indicate a buried interface area, a ΔG^diss^, and a number of potential hydrogen bonds and salt bridges across the interface (1825 Å^2^, 17.6 kcal/mol, 19 and 15 respectively) comparable to those calculated for *Ma*Pgb*.

The presence of a quaternary structure suggests that the protein may show coupling between the degree of ligation and affinity for the ligand. However, in view of i) the absence of effects due to the degree of photolysis on the CO rebinding kinetics and ii) the lack of any dependence of the CO binding kinetics on the fractional saturation of the dimer, we neglect any cooperative effect of the quaternary structure on the reaction scheme. This does not exclude that the quaternary structure may influence protein affinity for CO by affecting the tertiary structure of the monomers. Recent Molecular Dynamics investigations have shown that dimerization has profound consequences on the structure and the dynamics of *Ma*Pgb* [13]. The quaternary assembly mainly affects the spatial arrangement of helix G, which in turn influences the shape of tunnel 1.

The experimental evidence we have shown suggests that *Ma*Pgb* exists in two conformations, mainly characterized by different binding and, to a smaller extent, dissociation rates. Therefore, this conformational change appears to involve only the tertiary structure of each subunit without extending its effect to the partner subunit of the dimer. Ligation shifts the equilibrium towards a high affinity species (in the following indicated as MaPgb*^r^) with high binding and low dissociation rates. In the absence of the ligand, the protein adopts preferentially a lower affinity conformation (in the following indicated as MaPgb*^t^), with low binding and high dissociation rates. Nanosecond photolysis experiments demonstrate that, upon ligand dissociation, switching from the high to the low affinity conformation occurs on the sub-millisecond time scale.

In order to achieve a more quantitative description we have analyzed the CO binding kinetics using the kinetic model outlined in Scheme 1. The Scheme takes into account the existence of conformations that are in ligation-dependent equilibrium (ligation favours MaPgb*^r^, deligation favours MaPgb*^t^). Migration to a temporary docking site is included to account for transient binding to an internal cavity. This process occurs on a time scale over which the switch from MaPgb*^r^ to MaPgb*^t^ is negligible.
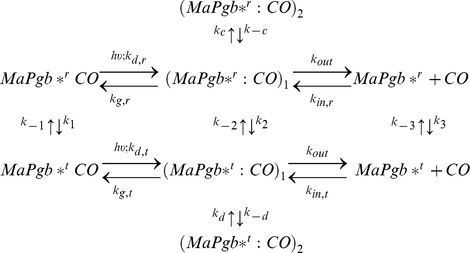



### Scheme 1

Minimal reaction scheme for the observed kinetics. After photodissociation of bound state, a mixture of MaPgb*^r^CO (≈70%) and MaPgb*^t^CO (≈30%), CO can migrate from the primary docking site (MaPgb*^r^:CO)_1_/(MaPgb*^t^:CO)_1_ to a secondary docking site (MaPgb*^r^:CO)_2_/(MaPgb*^t^:CO)_2_ or exit to the solvent (MaPgb*^r^ + CO)/(MaPgb*^t^ + CO). Rebinding occurs through two distinct pathways involving either MaPgb*^t^ or MaPgb*^r^. Different equilibrium constants connect MaPgb*^t^ with MaPgb*^r^ and MaPgb*^r^CO with MaPgb*^r^CO.

**Figure 7:**
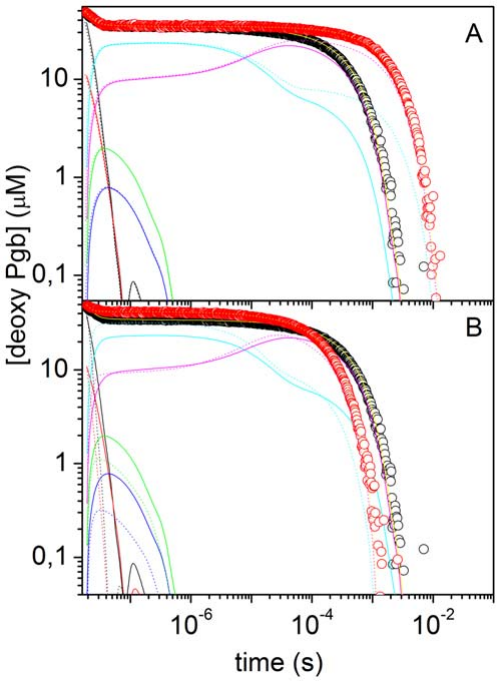
Representative analysis of CO rebinding kinetics to *Ma*Pgb*. (A) Analysis of the CO rebinding kinetics to *Ma*Pgb* in solution equilibrated at 10°C with 1 atm CO (black circles) and 0.1 atm CO (red circles). The fits (yellow lines) are superimposed to the experimental data (circles). In the figure we have also reported the time course of the other relevant species in Scheme 1 of main text: (MaPgb*^r^:CO)_1_ (black), (MaPgb*^t^:CO)_1_ (red), (MaPgb*^r^:CO)_2_ (green), (MaPgb*^t^:CO)_2_ (blue), (MaPgb*^r^) (cyan), (MaPgb*^t^) (magenta). Solid lines, 1 atm CO; dotted lines, 0.1 atm CO. (B) Analysis of the CO rebinding kinetics to *Ma*Pgb* in solution equilibrated with 1 atm CO at 10°C (black circles) and 25°C (red circles). The fits and the time courses of the other relevant species are also displayed with the same color code as in Panel A (solid lines, 10°C; dotted lines, 25°C).

Using Scheme 1 we were able to reproduce accurately the CO binding kinetics (in flash photolysis and stopped flow) for all tested experimental conditions (*Ma*Pgb* in solution, *Ma*Pgb* + CO gels, and CO*Ma*Pgb* gels). Other schemes failed to describe the body of experimental data we have accumulated. Figure 7 reports representative fits to CO rebinding kinetics to *Ma*Pgb* in solution after nanosecond laser photolysis, under selected experimental conditions. Besides showing the good agreement between the kinetic model and the experimental data, plots in Figure 7 also offer the time courses of the reaction intermediates, as detailed in the Figure caption. The complete list of microscopic rate constants at 20°C is given in Table 1, which also contains the activation free energies (*ΔG* ^‡^) at 20°C estimated from the activation enthalpies (*ΔH* ^‡^) and entropies (*ΔS* ^‡^) (Table S1).

As can be appreciated from the sample fits in Figure 7, migration to a temporary docking site occurs in low yield, and modulates the geminate phase in the tens of nanoseconds. The low efficiency of this reaction branch is due to comparatively high exit (*k_out_*) and rebinding (*k_g,r_*, *k_g,t_*) rates. Population of the temporary docking site is likely coupled to some structural rearrangement early on the reaction pathway. A spectroscopic trace of this fact is identified in the early time course of the amplitude of the second SVD component, reported in panel B of Figure 5. This may arise from conformational rearrangements of the aromatic residues surrounding the distal and the proximal sides, a process which may gate diffusion to the binding site. Given the short time scale over which these processes occur, it is unlikely that the location of the temporary docking site is far from the binding site. A possible candidate is the core cavity of about 75 Å^3^, located between the distal and proximal haem sides [6], although the presence of four mutually hydrogen-bonded water molecules in the crystal structure suggests that the photodissociated CO may not be easily accommodated.

The value of the exit rate *k_out_* is remarkably high, and demonstrates that the photodissociated ligand has a facile way out from the distal haem pocket, meaning that an open connections to the solvent is available, immediately after photodissociation of the ligand. The retrieved rebinding rates *k_in,r_*, *k_in,t_* are similar to those observed for other haemoglobins such as human neuroglobin [25], and plant non symbiotic haemoglobin 1 from *Arabidopsis thaliana* [23], [39], but larger than the values for human HbA [22], [40] and horse heart Mb [22].

Unlike other known haemoglobins, for which approximately 30% of the haem surface is solvent accessible, the haem in *Ma*Pgb* is completely shielded from the solvent, being surrounded by the distal B-, C- and E-helices, and by the proximal F-helix. This peculiar structural feature is related to the conformation of the 1–20 N-terminal segment, and to the extended CE and FG loops [6]. As a consequence of this three-dimensional outline, diatomic ligand diffusion to the haem pocket cannot exploit the well known distal His(E7)-gate, which is operative in Mb [41].

The two hydrophobic tunnels, identified in the three-dimensional structure [6], are the most likely candidates to support an efficient escape from the distal haem pocket and warrant high exit rate. A straight apolar protein matrix tunnel connects the protein surface to the haem distal cavity and is located between the B- and G-helices (tunnel 1). A second tunnel is located between the B- and the E-helices, and is partly defined by Tyr(B10)61 (tunnel 2). Access to neither of them appears to be restricted by the dimer contact surface. These tunnels thus provide direct connections between the haem distal pocket and the solvent in a similar fashion to what was recently reported for type 1 non symbiotic Hbs from *Arabidopsis thaliana* [23], [42] and rice [43].

The analysis of extended Molecular Dynamics simulations of *Ma*Pgb* dimers has shown that while tunnel 2 is always open, ligand accessibility through tunnel 1 may be regulated by Phe(145)G8, which can adopt open and closed conformations [13]. Dimerization and ligand binding strongly affect the ratio between open and closed states. Sensing of the ligand is mediated by Phe(93)E11, and the steric hindrance between Phe(93)E11 and the haem bound ligand alters the structural and dynamical behavior of helices B and E, which facilitates opening of tunnel 1.

Both Phe(145)G8 side-chain mobility and the ligand sensing properties of Phe(93)E11 suggest a structural basis to understand the nature of the fast and the slow rebinding conformations. In the open conformation, tunnel 1 leaves an additional open access from the solvent, thus resulting in higher probability (*k_in,r_*) for rebinding. At variance, for the unliganded state only tunnel 2 is open and the corresponding entry rate (*k_in,t_*) is lower. Investigations on mutants at positions E11 and G8 will provide key tests to understand the functional role of the residues Phe(93)E11 and Phe(145)G8.

At present it is difficult to fully understand the structural and dynamical basis for the change in Fe affinity for CO upon ligand binding. Further computational analysis and additional experimental evidence collected with mutated proteins is necessary to gain insight into the role of specific interactions between amino acid residues in the distal pocket in tuning association and dissociation rate constants.

It is worth comparing the apparent rate constants for binding (*k*_ON_) using the retrieved microscopic rates for the two pathways, *k_ON,t_∼k_in,t_k_g,t_*/(*k_g,t_+k_out_*), *k_ON,r_∼k_in,r_k_g,r_*/(*k_g,r_+k_out_*) with those determined from stopped flow experiments. The values we obtain are *k_ON,r_* = 2.1×10^7^ M^−1^ s^−1^ and *k_ON,t_* = 1.1×10^6^ M^−1^ s^−1^, and compare very well with those determined by stopped flow.

**Figure 8:**
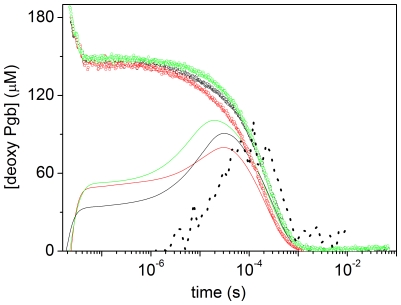
Structural relaxation from time resolved spectra and numerical analysis of CO rebinding. Comparison between the CO rebinding kinetics (circles) and the time courses of MaPgb*^t^ (solid lines) as retrieved from the numerical analysis after photolysis of CO*Ma*Pgb* solutions (black), CO*Ma*Pgb* gels (red), and *Ma*Pgb* + CO gels (green). Curves were measured at T = 20°C and 1 atm CO. The black dotted curve is the time evolution of the second spectral component obtained from the SVD analysis on transient spectra (see Figure 5).

On the microsecond time scales, the photodissociated protein MaPgb* ^r^ relaxes with rate *k*_3_ towards the lower affinity state MaPgb* ^t^, a process which is competitive with rebinding of CO from the solvent. Given the high rebinding rates (*k_g,r_*, *k_g,t_*, *k_in,r_* and *k_in,t_*), and the back reaction rate *k*_−3_, relaxation populates the slow reacting species to nearly 40–50% of the photodissociated molecules (i.e. MaPgb* ^r^ 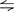 MaPgb* ^t^ does not fully relax to equilibrium before CO rebinding occurs,). This can be visually appreciated in Figure 8, where the concentration of MaPgb* ^t^ is plotted as a function of time (magenta curves). The microscopic rates *k*_−3_ and *k*_3_ define an equilibrium constant of 3 for the reaction MaPgb* ^r^ 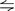 MaPgb* ^t^, showing that at equilibrium a substantial fraction (more than 70%) of the deoxy molecules are in the slow rebinding conformation MaPgb* ^t^. An identical equilibrium exists between (MaPgb*^r^:CO)_1_ and (MaPgb*^t^:CO)_1_ (equilibrium constant *k*_2_/*k*_−2_ = 2.4). The estimated equilibrium constant affords populations of fast (25%) and slow (75%) rebinding species which are roughly in agreement with the population of the fast and slow rebinding species observed in stopped flow experiments.

The corresponding equilibrium for the internal rates *k*_−1_ and *k*_1_ is more difficult to assess but suggests that COMaPgb* ^r^ is favored over COMaPgb* ^t^ in the presence of the ligand (equilibrium constant *k*_1_/*k*_−1_ = 0.3). Consistently, this figure compares well with the equilibrium constant determined from the fractions of slow and fast phases in the CO dissociation kinetics (0.49).

Relaxation of the equilibrium between species MaPgb*^r^ and MaPgb*^t^ can be visually appreciated in Figure 7. The magenta lines clearly show that MaPgb*^t^ is formed to a larger extent when the CO concentration is decreased (Figure 7A) and that formation and decay of this species is a thermally activated process (both forward and reverse reactions become faster as temperature is increased, Figure 7B).

Gel experiments allow to slightly but significantly affect the equilibrium between MaPgb*^r^ and MaPgb*^t^. In comparison to the experiments in solution (for which *k*_3_/*k*_−3_ = 3), relaxation of MaPgb*^r^ towards MaPgb*^t^ is kinetically favored in MaPgb*+CO gels, with the rate *k*_3_ increasing to 10^5^ s^−1^ (and the equilibrium constant to 4.4). On the contrary, for CO*Ma*Pgb* gels the equilibrium is slightly shifted towards MaPgb*^r^, the equilibrium constant decreasing to 2.8.

This effect can be clearly distinguished in Figure 8, where the time course of MaPgb*^t^ is shown for *Ma*Pgb* solutions, CO*Ma*Pgb* gels and *Ma*Pgb* + CO gels. The combination of rate constants is such that MaPgb*^t^ is formed to a maximum extent after photolysis of *Ma*Pgb* + CO gels, while MaPgb*^t^ is formed with the lowest efficiency for CO*Ma*Pgb* gels. The case of CO*Ma*Pgb* solutions appears as an intermediate case.

Figure 8 also compares the time courses of MaPgb*^t^ with the time evolution of the amplitude of the second spectral component retrieved from the SVD analysis (already reported in Figure 5). The striking similarity of the time courses is a strong indication that the spectral change obtained from the time resolved spectra reflects the structural relaxation leading the protein from MaPgb*^r^ to MaPgb*^t^. A non perfect matching between the black dotted curve and the progress curve for MaPgb*^t^ retrieved for CO*Ma*Pgb* solutions most likely arises from a maybe too drastic assumption that structural relaxation is a purely exponential process. Fitting of the second SVD component is best obtained with a sum of stretching exponential relaxations (Figure S5). This supports the idea that the structural relaxation is extended in time in a similar fashion to what is observed for Mb [44], [45], [46], [47] and human HbA [48].

The substantial free energy barriers for the forward and reverse rates of the tertiary relaxation are mostly entropic, with only modest (*k*_−3_ and *k*_3_) or negligible (*k*_−1_ and *k*_1_) enthalpic contributions (Table S1). This is an indication that the conformational change does not involve major rearrangements of the structure. For comparison, coordination (and dissociation) of the haem iron by the distal His, a process involving substantial molecular reshaping, has enthalpic barriers on the order of 14.1±0.9 kcal/mol (17±3 kcal/mol for the dissociation) for human Cygb [49] and 13 kcal/mol (18 kcal/mol for the dissociation) for human Ngb [50].

As expected, free energy barriers indicate that MaPgb*^t^ is more stable than MaPgb*^r^, while COMaPgb*^r^ is more stable than COMaPgb*^t^.

**Figure 9:**
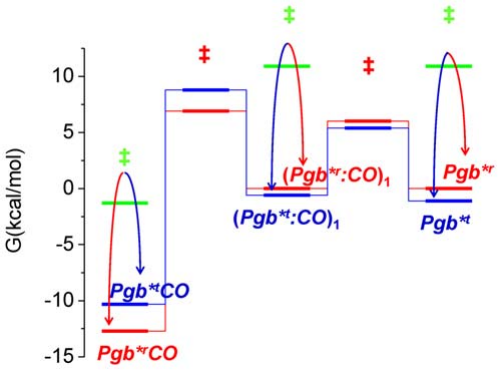
Free energy plot. The free energy plot for reaction Scheme 1 as calculated from activation free energies for the forward and reverse rate constants reported in Table 1. Transition states are labelled as ‡. Transitions between *r* and *t* conformations are indicated from curves with arrows. Solid lines (red, *r*; blue, *t*) connect states within each conformation.

Activation energies for the forward and reverse rates allow to build a free energy diagram for the reaction scheme (Figure 9). The barrier between MaPgb*^t^ and (MaPgb*^t^ *:CO*)_1_ is larger than the corresponding barrier separating MaPgb*^r^ and (MaPgb*^r^:CO)_1_, accounting for the higher second order rebinding rate observed for the latter reaction. The free energies of the unliganded species are lower for MaPgb*^t^ than for MaPgb*^r^, in agreement with the idea that in the absence of an exogenous ligand the protein spontaneously relaxes towards the conformation with lower binding and higher dissociation rates, resulting in lower affinity for CO. The reverse occurs for the liganded species.

### Conclusions

The heterogenous kinetics for ligand binding to and dissociation from *Ma*Pgb* observed in stopped flow and nanosecond laser flash photolysis experiments arises from a ligation-dependent equilibrium between a fast and a slow-rebinding conformation, with ligation favouring the former. As measured from laser flash photolysis experiments, the relaxation from the fast to the slow rebinding conformation occurs on the microsecond time scale. Encapsulation in silica gel partially allows for the selective freezing of one of the two conformations, suggesting a fairly large conformational difference. RR spectroscopy confirms the presence of more than one species in equilibrium, both in the unliganded and the liganded forms.

While our experiments dealt with the model gas CO, the change in rate constants for CO binding to and dissociation from the haem upon ligation/deligation may have consequences for a yet unknown reaction, sequentially involving two substrates.

This ligand-assisted regulation of protein affinity may be an ancient relic of an allosteric mechanism devised early along haemoglobin evolution.

## Supporting Information

Figure S1 **Resonance Raman spectrum of** ***Ma*** Pgb* in the CO-bound form. The excitation wavelength was 413.1 nm, laser power employed was 100 mW. Detail of the low-frequency (A) and high-frequency (B) region of the RR spectrum of CO-bound ferrous *Ma*Pgb* recorded at 100 mW laser power. The photolysis effect can be used to identify the Fe-His stretching mode. This low-frequency mode is only visible for pentacoordinated deoxy ferrous haem proteins and will increase upon increased photolysis. As it is evident from Figure S1B, due to photolysis of the haem-bound CO, most of the haem is in a deoxy ferrous state when a laser power of 100 mW is applied. In the low-frequency part of the spectrum (Figure S1A) a band can be observed in the ν_Fe-His_ region at 219 cm^−1^. This band depends on the laser power and can thus be assigned to the ν_Fe-His_ mode. The frequency is comparable to that of sperm whaleMb (220 cm^−1^) and Barley Hb (219 cm^−1^). The value of this mode indicates that the proximal histidine in penta-coordinated deoxy ferrous *Ma*Pgb* has an uncharged imidazole character and the haem is tightly bound to the proximal histidine. The frequency is quite close to that of the R-state in human Hb (222 cm^−1^), indicating a relatively unstrained proximal histidine.
(TIF)

Figure S2 **Analytical gel filtration experiments.** Dependence of the molecular mass on elution time; the the globin standards (open circles) were human haemoglobin (HbA), human cytoglobin (Cygb), bovine serum albumin (BSA), horse heart myoglobin (Mb) and cytochrome *c* (cyt *c*) along with Fe^3+^ *Ma*Pgb* (star).
(TIF)

Figure S3 **Bimolecular CO rebinding kinetics to** ***Ma*** Pgb*. Bimolecular CO rebinding kinetics to *Ma*Pgb* after laser flash photolysis of solutions equilibrated with CO at 1 atm (green) and 0.1 atm (blue). Red curves are the best fits to a sum of two exponential decay functions. T = 25°C.
(TIF)

Figure S4 **Results of biexponential decay fitting of second order CO rebinding.** Relative amplitudes (left) and lifetimes (right) of the two exponential decay functions (squares: fast reaction; circles: slow reaction) retrieved from the analysis of CO rebinding kinetics to *Ma*Pgb* in solution at 20°C at 1 atm (green) and 0.1 atm CO (blue), as a function of temperature.
(TIF)

Figure S5 **Analysis of time course of the second SVD component.** Fitting of the second component retrieved from the SVD analysis of the time resolved spectra following photolysis of a CO*Ma*Pgb* solution using a sum of three stretched exponential relaxations with lifetimes (stretching exponents, amplitudes) τ_1_ = 5 ns (β_1_ = 0.69, A_1_ = −0.22), τ_2_ = 99 µs (β_2_ = 0.48, A_2_ = −0.26), and τ_3_ = 390 µs (β_3_ = 1, A_3_ = 0.22). The stretched nature of the first and the second exponential relaxations demonstrate that the transition from MaPgb*^r^ to MaPgb*^t^ is extended in time and cannot be described to full accuracy by chemical kinetics as in Scheme 1. On the other hand, kinetics of the back relaxation from MaPgb*^r^ to MaPgb*^t^ is limited by the second order rebinding of CO, and as such is not showing extended nature. Accordingly, the last exponential decay is not stretched (β_3_ = 1).
(TIF)

Figure S6 **Lifetime distributions for CO rebinding after laser photolysis of CO** ***Ma*** Pgb* gels and ***Ma*** Pgb*+CO gels. Comparison between the lifetime distributions retrieved from fitting with the MEM analysis the CO rebinding kinetics after photolysis of *Ma*Pgb* gels soaked in a buffered solution. (A) *Ma*Pgb*+CO gels. (B) CO*Ma*Pgb* gels. Black curve, 0.1 atm CO; green curve, 1 atm CO. *T* = 20°C.
(TIF)

Table S1 **Microscopic rate constants for** ***Ma*** Pgb* from the fit of the flash photolysis data, at 20°C.
(DOC)
