# Supplementary figures and images for: Ligation Tunes Protein Reactivity in an Ancient Haemoglobin: Kinetic Evidence for an Allosteric Mechanism in Methanosarcina acetivorans Protoglobin
Source: PLoS One. 2012 Mar 27;7(3):e33614. doi: 10.1371/journal.pone.0033614 (PMC3313925; doi:10.1371/journal.pone.0033614)

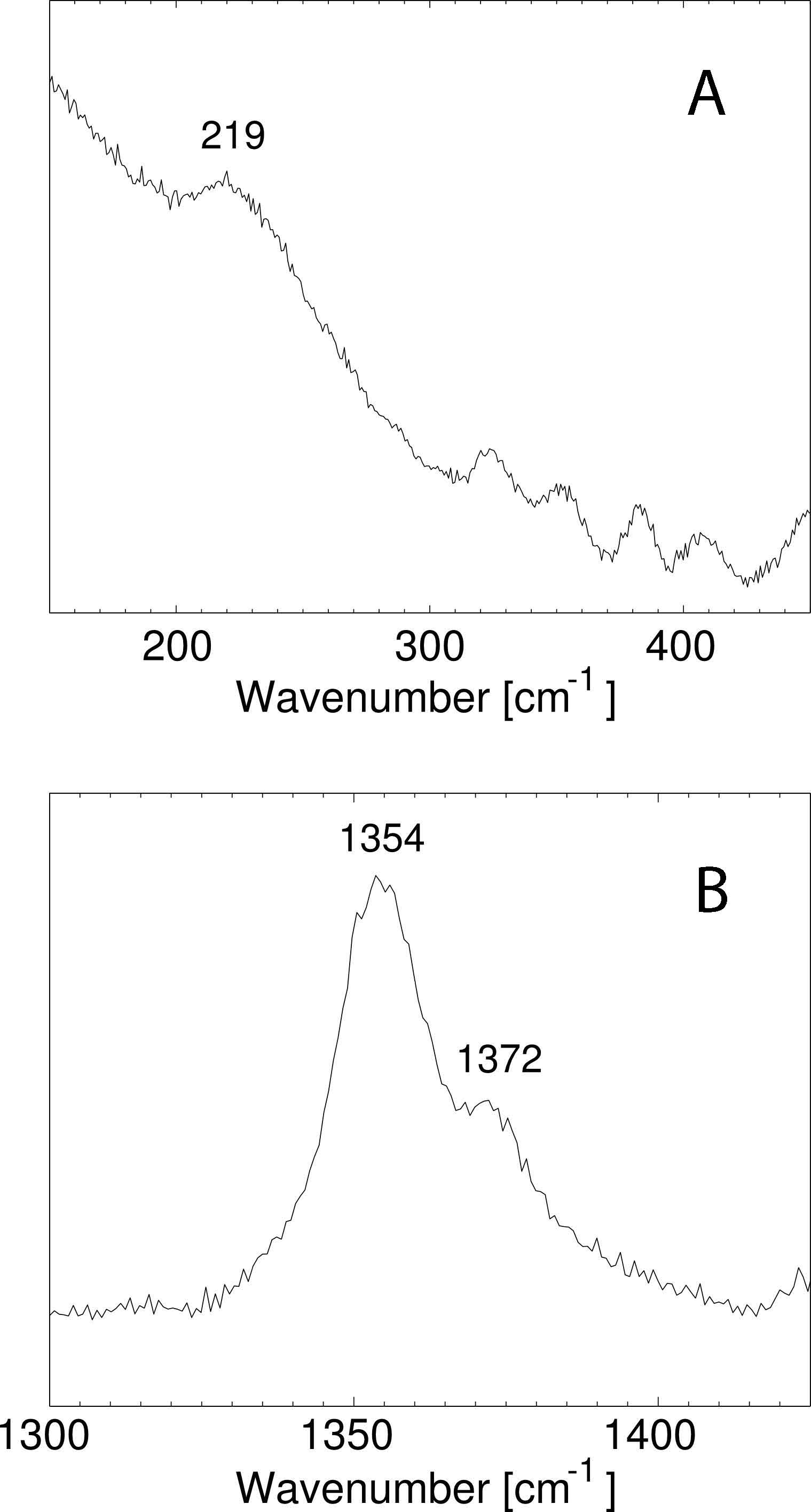

Supplement: Figure S1 — Resonance Raman spectrum of Ma Pgb* in the CO-bound form. The excitation wavelength was 413.1 nm, laser power employed was 100 mW. Detail of the low-frequency (A) and high-frequency (B) region of the RR spectrum of CO-bound ferrous MaPgb* recorded at 100 mW laser power. The photolysis effect can be used to identify the Fe-His stretching mode. This low-frequency mode is only visible for pentacoordinated deoxy ferrous haem proteins and will increase upon increased photolysis. As it is evident from Figure S1B, due to photolysis of the haem-bound CO, most of the haem is in a deoxy ferrous state when a laser power of 100 mW is applied. In the low-frequency part of the spectrum (Figure S1A) a band can be observed in the νFe-His region at 219 cm−1. This band depends on the laser power and can thus be assigned to the νFe-His mode. The frequency is comparable to that of sperm whaleMb (220 cm−1) and Barley Hb (219 cm−1). The value of this mode indicates that the proximal histidine in penta-coordinated deoxy ferrous MaPgb* has an uncharged imidazole character and the haem is tightly bound to the proximal histidine. The frequency is quite close to that of the R-state in human Hb (222 cm−1), indicating a relatively unstrained proximal histidine. (TIF) [file pone.0033614.s001.tif]

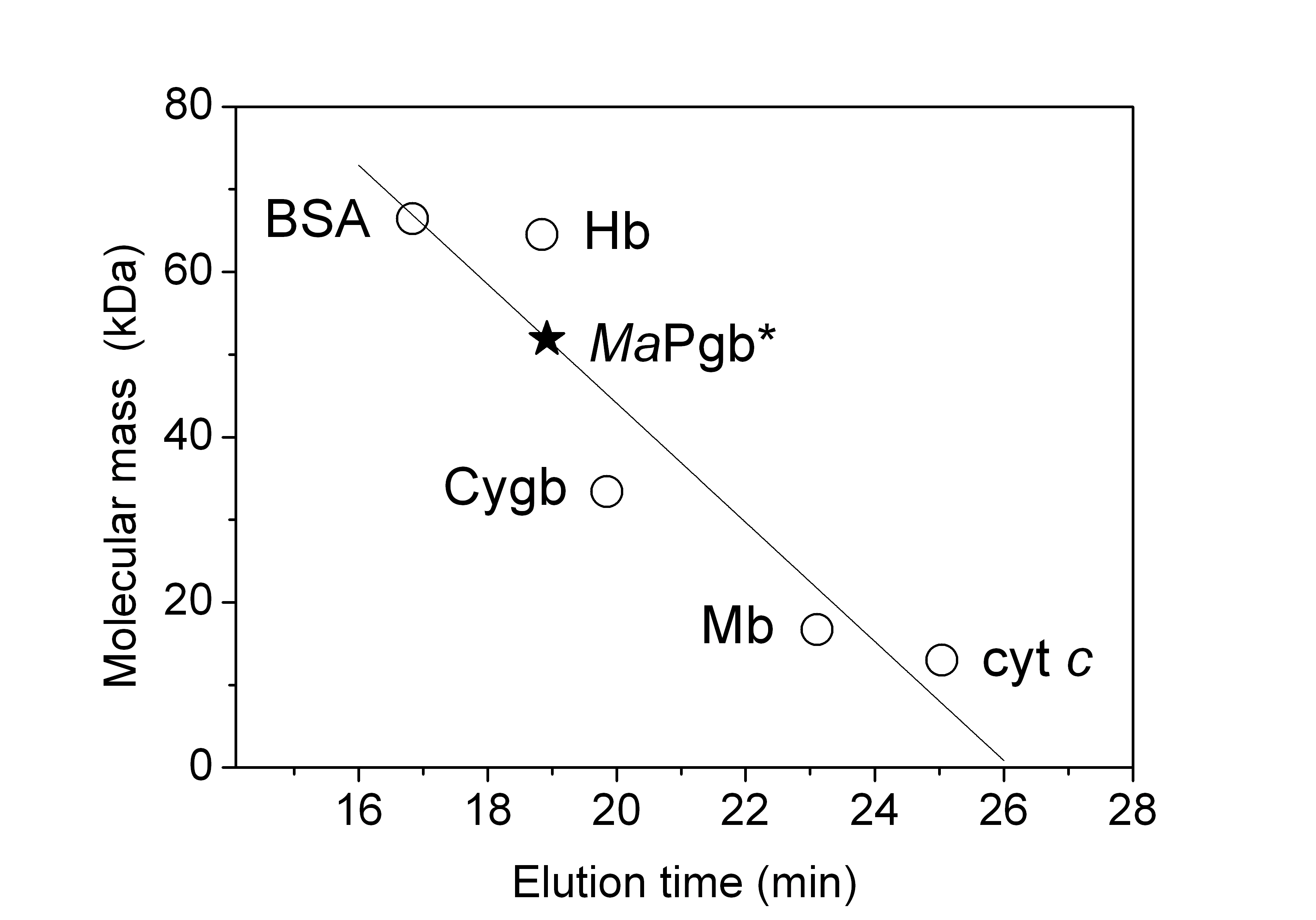

Supplement: Figure S2 — Analytical gel filtration experiments. Dependence of the molecular mass on elution time; the the globin standards (open circles) were human haemoglobin (HbA), human cytoglobin (Cygb), bovine serum albumin (BSA), horse heart myoglobin (Mb) and cytochrome c (cyt c) along with Fe3+ MaPgb* (star). (TIF) [file pone.0033614.s002.tif]
