# Supplementary material for: Ligation Tunes Protein Reactivity in an Ancient Haemoglobin: Kinetic Evidence for an Allosteric Mechanism in Methanosarcina acetivorans Protoglobin
Source: PLoS One. 2012 Mar 27;7(3):e33614. doi: 10.1371/journal.pone.0033614 (PMC3313925; doi:10.1371/journal.pone.0033614)
Supplement: Table S1 — Microscopic rate constants for Ma Pgb* from the fit of the flash photolysis data, at 20°C. (DOC) [file pone.0033614.s007.doc]

**Table S1**

Microscopic rate constants for *Ma*Pgb* from the fit of the flash photolysis data, at 20 °C. Activation free enthalpies and entropies were estimated from the linear Eyring plots for each rate constant *k*_i_ in the temperature range 10-30 °C. The Gibb’s activation free energies at 20 °C are also reported.

|  |  | **solution** |  |  |  | **COPgb* gel** |  |  |  | **(CO+Pgb*) gel** |  |  |
| --- | --- | --- | --- | --- | --- | --- | --- | --- | --- | --- | --- | --- |
|  | k | *ΔS*^‡^(cal/molK) | *ΔH*^‡^(kcal/mol) | *ΔG*^‡^(kcal/mol) | *k* | *ΔS*^‡^(cal/molK) | *ΔH*^‡^(kcal/mol) | *ΔG*^‡^(kcal/mol) | *k* | *ΔS*^‡^(cal/mol K) | *ΔH*^‡^  (kcal/mol) | *ΔG*^‡^(kcal/mol) |
| *k*_1_ (10^5^ s^-1^) | 1.35 | -35.0 ± 0.1 | - | 10.51±0.1 | 1.35 | -35.0± 0.06 | - | 10.5±0.02 | 1.35 | -35 ± 0.1 | - | 10.52±0.02 |
| *k*_-1_(10^5^ s^-1^) | 4.55 | -32.6 ± 0.1 | - | 9.8±0.1 | 4.55 | -32.6 ±0.06 | - | 9.8±0.1 | 4.5 | -32 ± 0.1 | - | 9.8±0.02 |
| *k*_2_(10^5^s^-1^) | 0.6 | -33.2 ± 0.4 | 1.0 ± 0.1 | 10.9±0.2 | 0.4 | -13.7 ± 0.6 | 6.9± 0.2 | 11.1±0.4 | 1 | -3 ± 1 | 9.4 ± 0.3 | 10.5±0.6 |
| *k*_-2_ (10^4^s^-1^) | 2.5 | -26 ± 1 | 3.6 ± 0.4 | 11±1 | 1.4 | -21.3 ± 0.1 | 5.3 ± 0.1 | 11.7±0.1 | 2.5 | -38.3 ± 0.1 | - | 11.49±0.03 |
| *k*_3_ (10^5^ s^-1^) | 0.6 | -33.2 ± 0.5 | 0.9 ± 0.1 | 10.9±0.2 | 0.4 | -13 ±1 | 7.1 ± 0.4 | 11.0±0.7 | 1.1 | -7 ± 3 | 8.2 ± 0.9 | 10±2 |
| *k-*_3_ (10^4^s^-1^) | 2.0 | -24 ± 2 | 4.4 ± 0.5 | 11.5±1.1 | 1.4 | -21 ± 1 | 5.3± 0.2 | 11.7±0.4 | 2.5 | -38.3 ± 0.1 | 4 ± 1 | 11.48±0.03 |
| *k_in,r_* (10^7^ M^-1^s^-1^) | 7.8 | 27 ± 4 | 14 ± 1 | 6±2 | 7.2 | -27 ± 5 | 15 ± 1 | 6±3 | 6.9 | 28 ± 3 | 14 ± 1 | 6±2 |
| *k_in,t_* (10^7^M^-1^s^-1^) | 3 | 12 ± 9 | 11 ± 2 | 7±3 | 1 | 138 ± 16 | 48 ± 1 | 6.8±1 | 1 | 66 ± 3 | 27 ± 1 | 7±2 |
| *k_out_* (10^8^s^-1^) | 1.5 | 12 ± 2 | 10 ± 1 | 6±1 | 1.4 | 15 ± 7 | 11 ± 2 | 6±4 | 1 | 10 ± 5 | 9 ± 1 | 6±3 |
| *k_g,r_* (10^7^ s^-1^) | 5.5 | -23.1± 0.1 | - | 6.9±0.1 | 5.3 | -23.6 ± 0.3 | - | 7.1±0.1 | 3.8 | -23.8 ± 0.1 | - | 7.16±0.02 |
| *k_g,t_* (10^6^ s^-1^) | 6 | -27.5 ± 0.1 | - | 8.2±0.1 | 3.8 | -28.2 ± 0.5 | - | 8.4±0.1 | 3.8 | -28.4 ± 0.1 | - | 8.53±0.02 |
| *k_d,r_* (10^-2^ s^-1^) | 4.4 | -65.5± 0.1 | - | 19.6±0.1 | 4.4 | -65.5±0.1 | - | 19.6±0.1 | 4.4 | -65.5 ± 0.1 | - | 19.65±0.02 |
| *k_d,t_* (10^-2^ s^-1^) | 8.4 | -63.5 ± 0.1 | - | 19.1±0.1 | 8.3 | -63.5 ± 0.1 | - | 19.0±0.1 | 8.4 | -63.5 ± 0.1 | - | 19.06±0.02 |
| *k*_c_ (10^7^ s^-1^)* | 1 | -26.5± 0.1 | - | 8.0±0.1 | 1 | -29± 2 | - | 8.7±0.7 | 1 | -26.5 ± 0.1 | - | 7.95±0.02 |
| *k-*_c_ (10^7^ s^-1^)* | 1 | -26.5± 0.1 | - | 8.0±0.1 | 0.7 | -26.8± 0.3 | - | 8.0±0.1 | 0.9 | -26.5 ± 0.1 | - | 7.95±0.02 |
| *k*_d_ (10^7^ s^-1^)* | 1 | -26.5± 0.1 | - | 8.0±0.1 | 1 | -29± 3 | - | 8.8±0.9 | 0.6 | -32.1 ± 0.1 | - | 9.6±0.02 |
| *k*_-d_ (10^7^ s^-1^)* | 1 | -26.5± 0.1 | - | 8.0±0.1 | 0.7 | -26.6± 0.4 | - | 7.9±0.1 | 1.1 | -26.3 ± 0.1 | - | 7.89±0.02 |
